# Supplementary material for: Highly Efficient n-Type Doping of Graphene by Vacuum Annealed Amine-Rich Macromolecules
Source: Materials (Basel). 2020 May 8;13(9):2166. doi: 10.3390/ma13092166 (PMC7254272; doi:10.3390/ma13092166)
Supplement: Supplementary file 1 [file materials-13-02166-s001.pdf]

materials

Supplementary Material

Highly Efficient *n*-type Doping of Graphene by Vacuum Annealed Amine-rich Macromolecules

Young-Min Seo [1], Wonseok Jang [1], Taejun Gu [1] and Dongmok Whang [1,2] *

[1] School of Advanced Materials Science & Engineering, Sungkyunkwan University, Suwon 16419, Korea; black4504@skku.edu (Y.-M.S.); wid0129@skku.edu (W.J.); gtj1008@skku.edu (T.G.)
[2] SKKU Advanced Institute of Nanotechnology (SAINT), Sungkyunkwan University, Suwon 16419, Korea
* Correspondence: dwhang@skku.edu

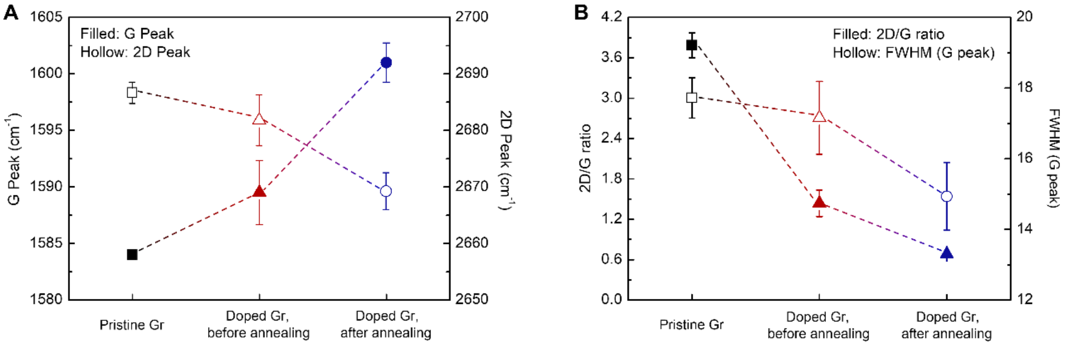

Figure S1. Raman spectra of graphene before/after doping&vacuum annealing. (**A**) Position of G and 2D peak of each graphene. (**B**) 2D/G ratio and full width half maximum (FWHM) of G peak of each graphene.

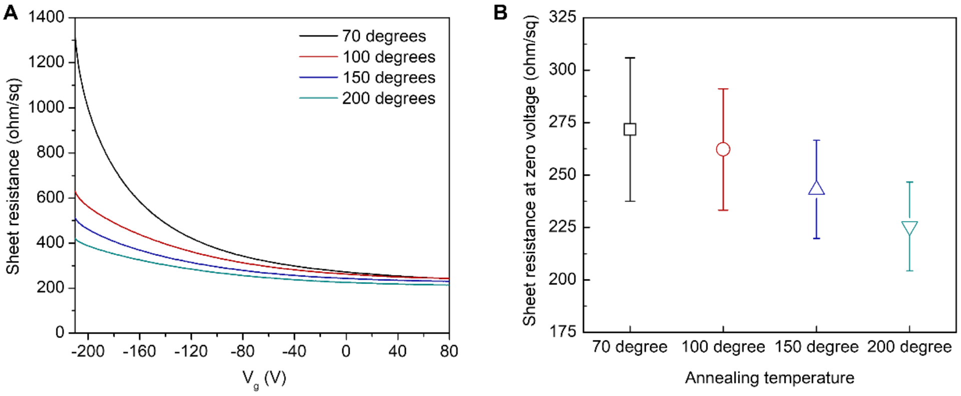

Figure S2. Changes in GFETs characteristics according to annealing temperature. (**A**) Gate-dependent electrical property of PEI doped GFETs characteristics according to annealing temperature. (**B**) Changes in sheet resistance of PEI doped GFETs according to annealing temperature.

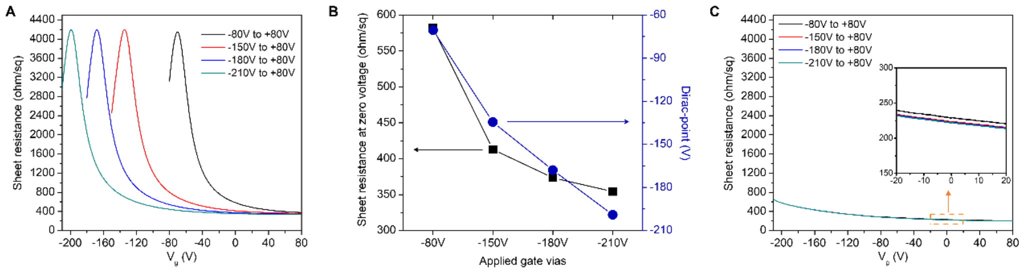

Figure S3. Changes in GFETs characteristics according to gate voltage before and after vacuum annealing. (**A**) Gate-dependent electrical property of PEI doped GFETs characteristics depending on the gate sweep range before annealing. (**B**) Changes in sheet resistance and Dirac-point of GFETs by applied gate voltage. (**C**) Gate-dependent electrical property of PEI doped GFETs characteristics depending on the gate sweep range after annealing. Inset is enlarged graph of the orange dash line.

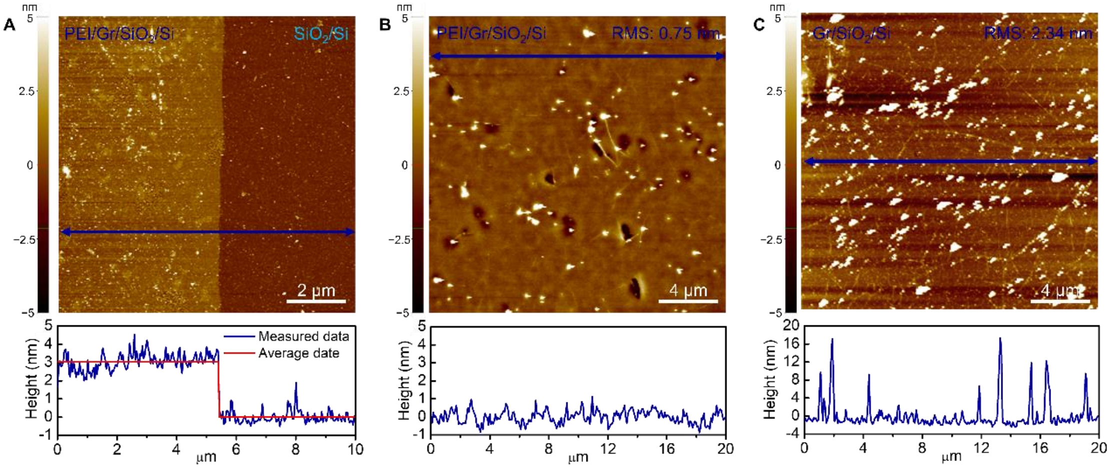

Figure S4. Atomic force microscope (AFM) analysis of surface height and uniformity. Each graph under the images are surface roughness of blue arrow lines on the image. (**A**) Thickness of PEI/Gr structure on SiO_2/Si b. Since the thickness of graphene is 0.3 nm, the thickness of PEI is less than 3 nm. (**B**) Roughness of PEI /Gr/SiO_2/Si. (**C**) Roughness of Gr on SiO_2/Si.

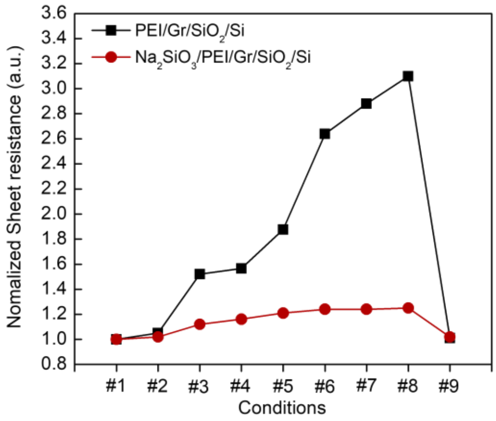

Figure S5. Sheet resistance change in air atmosphere before and after passivation of PEI doped GFET. Sodium metasilicate used as a passivation layer was diluted (5 wt %) in Di, spin-coated (3000 rpm) on PEI/Gr/SiO_2/Si and baked on a hot plate for 120 °C and 20 min to form a film. #1 condition was measured on vacuum and #2, 3, 4~8 was 1 h, 24 h, 48 h, 72 h, 7 days, 10 days, 14 days later respectively. #9 was after re-annealed each devices at 150 °C, for 2 h.
